# Social Learning as a Way to Overcome Choice-Induced Preferences? Insights from Humans and Rhesus Macaques

**DOI:** 10.3389/fnins.2012.00127

**Published:** 2012-09-03

**Authors:** Elisabetta Monfardini, Valérie Gaveau, Driss Boussaoud, Fadila Hadj-Bouziane, Martine Meunier

**Affiliations:** ^1^INSERM, U1028, ImpAct Team, Lyon Neuroscience Research CenterLyon, France; ^2^CNRS, UMR5292, ImpAct Team, Lyon Neuroscience Research CenterLyon, France; ^3^University LyonLyon, France; ^4^Institut de Médecine EnvironnementaleParis, France; ^5^Institut de Neuroscience des Systèmes, UMR 1106, INSERM, Aix-Marseille UniversitéMarseille, France

**Keywords:** social learning, reinforcement learning, cognitive biases, choice-induced preference, rhesus macaques, humans

## Abstract

Much theoretical attention is currently devoted to social learning. Yet, empirical studies formally comparing its effectiveness relative to individual learning are rare. Here, we focus on free choice, which is at the heart of individual reward-based learning, but absent in social learning. Choosing among two equally valued options is known to create a preference for the selected option in both humans and monkeys. We thus surmised that social learning should be more helpful when choice-induced preferences retard individual learning than when they optimize it. To test this prediction, the same task requiring to find which among two items concealed a reward was applied to rhesus macaques and humans. The initial trial was individual or social, rewarded or unrewarded. Learning was assessed on the second trial. Choice-induced preference strongly affected individual learning. Monkeys and humans performed much more poorly after an initial negative choice than after an initial positive choice. Comparison with social learning verified our prediction. For negative outcome, social learning surpassed or at least equaled individual learning in all subjects. For positive outcome, the predicted superiority of individual learning did occur in a majority of subjects (5/6 monkeys and 6/12 humans). A minority kept learning better socially though, perhaps due to a more dominant/aggressive attitude toward peers. Poor learning from errors due to over-valuation of personal choices is among the decision-making biases shared by humans and animals. The present study suggests that choice-immune social learning may help curbing this potentially harmful tendency. Learning from successes is an easier path. The present data suggest that whether one tends to walk it alone or with a peer’s help might depend on the social dynamics within the actor/observer dyad.

## Introduction

Social species, like rhesus macaques and humans, have two main ways of coping with novel problems. They can either learn to distinguish between good and bad choices individually (i.e., through their own experience) or, alternatively, they can rely on social learning (Miller and Dollard, 1941). Which of these two forms of learning is more effective? This question has received a substantial amount of theoretical attention, from both psychology and neighboring disciplines such as ethology and economics. Most models argue that the remarkable ability to learn from others’ successes and failures not only saves effort and time, but also allows cultural knowledge to accumulate over generations (Bandura, 1977; Cavalli-Sforza and Feldman, 1981; Boyd and Richerson, 1985). Other theoretical works, however, have reached the conclusion that copying others is not *per se* a recipe for success (e.g., Laland, 2004; Valone, 2007; Rieucau and Giraldeau, 2011). These models emphasize the prevalence of trade-offs in the use of social and personal information and suggest that subjects exploit socially transmitted information only where individual learning would be risky or impossible (e.g., learning to escape predators; Giraldeau et al., 2002; Kendal et al., 2005).

At the empirical level, several recent studies have provided convincing evidence that trial-and-error reward-based learning is faster in monkeys and humans when it is preceded by observation of an expert or novice conspecific (Brosnan and de Waal, 2004; Subiaul et al., 2004, 2007; Meunier et al., 2007). These studies, however, compared individual learning with vs. without prior observation of others. They did not attempt to determine whether the exact same amount of information yields better learning when acquired socially than when obtained individually. To our knowledge, only two studies, both carried out in humans, performed such controlled comparison of the effectiveness of social and individual learning and they led to opposite conclusions. Rendell et al. (2010) described a case where the most successful strategy for learning relied almost exclusively on copying. In contrast, Nicolle et al. (2011) reported a case where social learning was less effective than individual learning. In sum, despite the large amount of theoretical attention devoted to social learning benefits and pitfalls, controlled empirical evaluations of the relative effectiveness of social vs. individual learning remain rare, limited to humans, and of mixed results. The current study enriches this scarce empirical knowledge. Its aim was to identify a strong predictor of differences in relative effectiveness of these two forms of learning.

Literature to date has ignored a major difference between individual and social learning: free choice, which is at the very heart of feedback-based learning, is simply absent in social learning. Some variations in effectiveness between individual and social learning could stem from this major difference. The mere act of choosing between equally valued options *creates*, rather than reflects, a preference. In his seminal paper, Brehm (1956) presented participants with a set of daily-life articles and they were asked to rate each of them based on its desirability. After performing this rating, participants were given the choice between two of these objects previously evaluated as equally attractive. Subjects were then asked to rate each of the articles again. Results showed that, after making the difficult choice between two equally preferred alternatives, people tended to like the selected item more and the rejected item less than they originally did. Further studies amply confirmed this tendency in humans (e.g., Festinger and Carlsmith, 1959; Gerard and White, 1983; Jones, 1985; Ariely and Norton, 2008; Sharot et al., 2009, 2010; Izuma et al., 2010; Johansson et al., 2012), and one recent study demonstrated that it exists in monkeys as well (Egan et al., 2007; see also Egan et al., 2010).

This propensity to reevaluate the intrinsic value of choice alternatives, known in social psychology as choice-induced preference, also accounts for a robust learning bias classically observed in object discrimination learning. Faced with two equally neutral objects, one leading to a reward, the other not, individuals from several species (rhesus monkeys: Riopelle et al., 1954; Riopelle, 1955, 1960; Itoh et al., 2001; cats: Warren, 1959; humans: van Duijvenvoorde et al., 2008; baboons and pigeons: Cook and Fagot, 2009) are far less likely to learn if they happen to select the negative object during their initial choice than when they initially pick the positive object. When the initial choice proves incorrect, choice-induced attraction for the negative item considerably slows subsequent learning.

We therefore used an object discrimination task to test the idea that the effectiveness of social learning can be predicted by the influence of free choice on individual learning. Specifically, we surmised that when a choice-induced preference hinders individual learning (i.e., after initial selection of the negative item), social learning should prove advantageous; conversely, when a choice-induced preference eases individual learning (i.e., after initial selection of the positive item), social learning should prove inferior. The same task was applied to humans and rhesus macaques in order to demonstrate that this variation in social learning effectiveness is a phylogenetically ancient trend that arose over evolution and operates regardless of language, culture, and experience.

## Materials and Methods

### Subjects

#### Monkeys

Two groups, each comprising three captive-born rhesus macaques (*Macaca mulatta*), participated to the study. One group was composed of 4-year-old males, the other of 3-year-old females. Each group was tested in its usual living quarters. The male group lived in a large indoor/outdoor enclosure and was tested outdoors (see Meunier et al., 2007). The female group was laboratory-housed and was tested indoors in the communicating individual cages they shared. During testing, all three group members were present, each in a separate compartment, the two members playing the observer and actor roles being placed either at a 90° angle (male group) or face to face (female group). Each monkey was tested with the partner he/she was the most willing to work with. Monkeys were not food-deprived; they were fed after testing completion but received their normal food rations of fresh fruits and monkey chow. Water was always available. All procedures involving monkeys were in accordance with the European Community’s Council Directive for the Care and Use of Laboratory Animals (86/609/EEC).

#### Humans

Six subjects were recruited and asked to come with a companion of their choice. The study thus involved a total of 12 human subjects, eight males and six females (mean age ± SEM: 31 ± 2 years, range 24–53). Consent was obtained from each subject. All procedures involving humans were in accordance with the French Law (Titer I and II du Code de la Santé Publique).

### Apparatus

A test tray equipped with two food wells was used to confront monkeys and humans to pairs of objects. For the male monkey group (outdoor testing), the tray was hidden under a large bucket while the experimenter positioned the objects and reward. For the female monkey group (indoor testing), the tray was mounted on a wheeled cart equipped on each side with two screens, one opaque and one transparent, allowing the experimenter to control what the two animals situated on each side of the apparatus could see or do. For humans, the tray was mounted on a wheeled cart equipped with an opaque screen on one side; the experimenter sat on the open side of the apparatus while the observer and the actor sat side by side on the screened side. Note that subjects, monkeys and humans, were never tested alone; differences observed across conditions cannot therefore be attributed to the social facilitation/inhibition phenomenon triggered by the mere presence or absence of another’s subject, which is well-known in humans (Bond and Titus, 1983) and also exists in monkeys (Addessi and Visalberghi, 2001; Dindo et al., 2009).

### Task principle

Lists of several pairs of stimuli were used. The pairs composing the list were presented one after the other, always in the same order. Within each pair, one of the two objects always led to a positive outcome (a reward), the other always to a negative outcome (lack of reward). The left/right position of the positive object was pseudo-randomized across presentations. Monkeys were trained, and humans were asked, to displace only one of the two objects per trial and no correction procedure was applied after an error. Monkeys’ preliminary training was the same as in earlier studies using real objects (e.g., Meunier et al., 1993, 1997, 2007). It generally lasted 4–6 days and consisted in presenting first a single baited object until the animal readily displaced it to retrieve the candy, and then two objects one baited, one unbaited, until the animal understood that he/she was not allowed to displace the second object after an unrewarded first choice and that correct responses were determined by the object and not by its spatial location. Preliminary training was successful inasmuch as monkeys showed no spatial bias on the first testing session making 49.7 ± 0.2% of right-sided responses (one-sample *t*-test relative to 50%: *t*5 = 0.1, *p* = 0.90).

During testing, two types of trial 1, experienced and observed, were mixed within each list as detailed in the Procedure section below. In the first case (individual learning), the subject was given access to the test tray and could freely choose one between the two-presented items. In the second case (social learning), he/she was provided with the opportunity to observe and benefit from his/her companion’s choice. In both learning conditions, reward was manipulated unbeknownst to subjects so that, over the course of the experiment, the actor experienced and the model demonstrated an equal number of correct and incorrect choices on trial 1. A reward was concealed under both objects to obtain a correct trial 1, whereas neither object was rewarded to obtain an incorrect trial 1. This manipulation enforced a 50% chance performance on trial 1 in each learning condition.

Monkeys were not taught (and humans were not instructed) to observe their companion. The study relied for both species on subjects’ spontaneous willingness to observe a peer’s behavior. The percent correct response on trial 2 was compared across groups to determine the effectiveness of social learning vs. individual learning.

### Procedure

#### Monkeys

**Figure 1:**
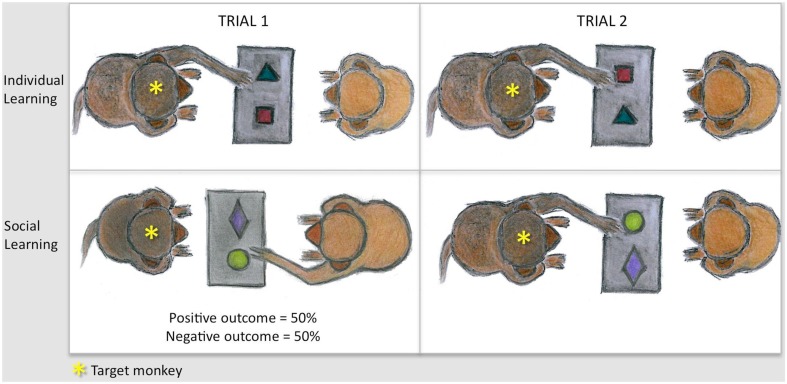
**Sketches illustrating the task principle**. Monkeys are shown but the principle was the same for humans. Subjects were tested in dyads on an object discrimination task. Pairs of objects were presented on a tray equipped with two food wells. Each pair contained a positive item and a negative one. The goal was to find the positive item to obtain the reward concealed underneath. Trial 1 (left panel) was either actively performed (individual learning, up) or passively witnessed (social learning, bottom). In both conditions, equal numbers of successes and errors were obtained by baiting both wells for half the trials and none of the wells for the other half. The relative effectiveness of individual vs. social learning was assessed, separately for each outcome, by measuring subjects’ percent correct responses on a second trial (right panel). Subjects actually learned lists of several object pairs in order to test all four types of trial 1 (individual/social, negative/positive) within each testing session (cf. Procedure in Material and Methods).

Stimuli were real objects (toys, cardboard boxes, plastic containers, etc.). Rewards were chocolate candies. Each list comprised nine pairs. Within each list, stimuli varied in shape, size, texture, and color. Each monkey learned a total of 10 lists over 10 separate sessions. A very large pool of objects was constituted to ensure that each individual saw a different list each time he/she participated as actor or model. For each session (Figure 1), the model first showed six pairs with three successes and three errors appearing in pseudo-random order. Then, three additional pairs were inserted in the list, and this now complete list was given to the actor. The actor monkey actually completed the full list 10 times, but only the first and second encounters with a pair (trials 1 and 2) will be considered in the present study.

#### Humans

At completion of the monkey experiment, a pilot study was carried out in humans. Stimuli and procedure were tailored to equate the level of difficulty across species. The main difficulty was to circumvent humans’ tendency to create verbal labels for stimulus-outcome associations and rehearse them during inter-trial intervals. Two changes were thus introduced relative to the monkey task. First, stimuli were computer-generated complex geometric patterns printed on 10 cm × 10 cm cardboard plaques. Second, humans were to search for the object concealing a reward (a 0.20€ coin) in each stimuli pair they were presented with and, at the same time, to listen to stories (tales from the “Just So Stories” by Rudyard Kipling) in order to answer a quiz at the end of the session, each question correctly answered earning an additional 0.20€ reward (total gains ranged from 15 to 25€ per volunteer depending on performance). Otherwise, human testing followed the same principle as monkey testing. Each list comprised eight pairs. Within each list, stimuli varied in shape, but were of the same overall size and color. Each human dyad learned a total of 20 different lists over two separate daily sessions, 10 lists per subject, five per day. Each list was used only once. For each session, the model first showed four pairs with two successes and two errors appearing in pseudo-random order. Then, four additional pairs were inserted either at the beginning or at the end of the list, and the now complete list was given to the actor twice. The study was presented as a memory experiment to the human volunteers and the request to come with a companion was explained away by the necessity to maintain the same testing conditions as in a parallel monkey study. Concealing the real aim of the experiment ensured that, like monkeys, humans watched their companion’s choices only if spontaneously compelled to do so.

### Social interactions evaluation

#### Monkeys

Social interactions within our two monkey trios were evaluated by testing each animal twice, once with each peer. During testing, the two animals were placed in the same compartment in presence of, but separated from the remaining group member. The first test consisted in delivering 10 food treats at equal distance from the two animals. The total number of eaten treats determined the animal’s rank, the monkey that ate the largest number of treats being attributed rank 1. The second snapshot of the social hierarchy within each group was obtained by videotaping dyadic interactions for 10 min after an overnight separation to promote social contacts. The duration of aggressive (e.g., receive/give chasing) and affiliative (e.g., receive/give grooming) behaviors were scored using Noldus software The Observer^®^XT10. A rank was attributed for each recorded behavior following known behavioral expressions of social status in macaques e.g., receiving the most grooming yielded rank 1 (Shively, 1998; Stavisky et al., 2001). In the present study, ranking was actually based on affiliation because aggression seldom occurred in the recorded samples. Then, the overall rank of each monkey was determined by averaging the ranks across all observed behaviors.

#### Humans

To probe interactions within our human dyads, we used a French questionnaire designed by Chalvin (1999, 2009). Subjects self-assessed their way of interacting with others by answering yes or no to 60 statements. Positive answers fell into four categories: assertive, passive, aggressive, or manipulative behaviors (score max per category = 15).

### Data analysis

Each monkey learned a total of 90 pairs, trial 1 being observed for 60 pairs (30 with positive outcome and 30 with negative outcome) and experienced for the remaining 30 pairs (15 with positive outcome and 15 with negative outcome). Each human learned a total of 80 pairs, trial 1 being experienced for 40 pairs (20 with positive outcome and 20 with negative outcome) and observed for the remaining 40 pairs (20 with positive outcome and 20 with negative outcome). Scores, i.e., the percent correct responses on trial 2, were calculated for individual and social learning, for each species (monkeys and humans) and for each outcome (positive and negative) and then analyzed using two learning × two species × two outcome ANOVAs and one-sample, two-sample, or paired *t*-tests as appropriate.

To quantify the advantage or disadvantage conferred by social learning over individual learning, we calculated two learning Δs (social score – individual score/individual score) per subject, one for each outcome. Positive Δs indicated that social learning was advantageous, negative Δs that individual learning was optimal. Then, Pearson’s correlations were performed, within and across species, on these learning Δs to determine whether they were best predicted by individual or by social learning scores, that is, whether social learning effectiveness varied with personal learning skills or, alternatively, depended on the social dynamics existing within the dyad.

## Results

### Individual learning

**Figure 2:**
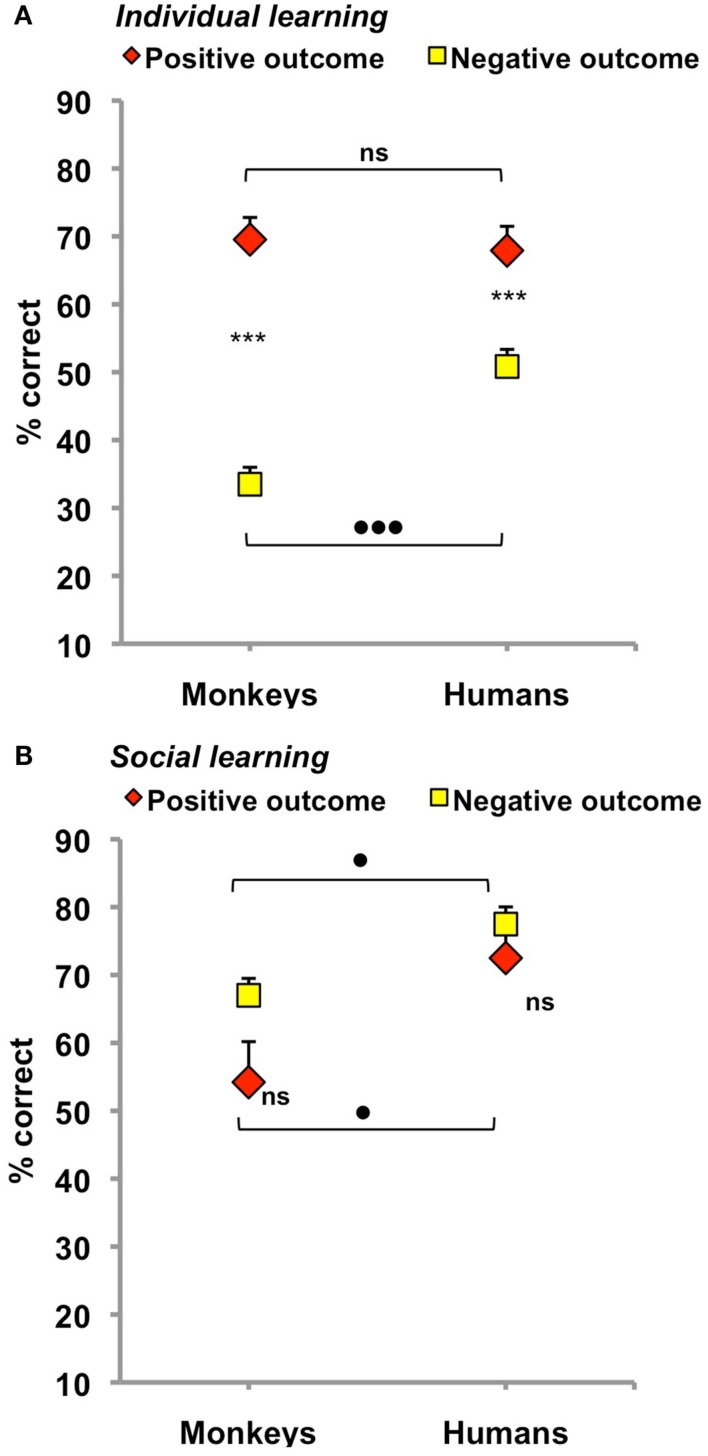
**Overall performance on trial 2 in monkeys (left) and humans (right) for individual (A) and social (B) learning after a single negative vs. positive outcome**. Scores are% correct responses (mean ± SEM). Asterisks denote within-species differences as revealed by paired *t*-tests, ***p < 0.001, black dots indicate between-species differences as revealed by two-sample *t*-tests. ^•^*p* ≤ 0.05, ^•••^*p* = 0.003. Note that both species learned best from positive outcome, individually, and from negative outcome, socially.

When trial 1 involved a choice (Figure 2A; Table 1), the two species achieved their best performance after a positive outcome [main effect of outcome: *F*(1,16) = 88.8, *p* < 0.001]. Monkeys and humans reached 70 and 68% correct responses, respectively, two statistically indistinguishable scores suggesting that task difficulty was successfully equated across species. Performance plummeted after a negative outcome and this decrease was larger in monkeys than in humans [main effect of species: *F*(1,16) = 5.9, *p* = 0.03; species × outcome interaction: *F*(1,16) = 11.3, *p* = 0.004]. Monkeys indeed managed only 33% correct responses after a negative outcome, a 37% loss relative to positive outcome, compared to 51% correct responses, and a 17% loss, for humans (see Figure 2A for *post hoc* comparisons).

**Table 1:** **Individual scores (% correct responses) and species averages obtained on trial 2 for individual and social learning after a single negative vs. positive outcome**.

Group	Gender	Negative		Positive	
		Individual	Social	Individual	Social
Dyad 1	Male	50	90	65	95
Dyad 1	Female	40	65	65	40
Dyad 2	Male	50	90	65	95
Dyad 2	Male	40	95	65	40
Dyad 3	Female	50	75	60	80
Dyad 3	Female	50	85	80	80
Dyad 4	Male	50	75	60	80
Dyad 4	Male	55	80	80	70
Dyad 5	Female	40	75	65	85
Dyad 5	Male	60	65	85	85
Dyad 6	Female	70	70	65	75
Dyad 6	Female	55	65	60	45
**Humans**	**Average**	**51**	**78**	**68**	**73**
Trio 1	Male	24	68	67	77
Trio 1	Male	39	71	83	59
Trio 1	Male	42	83	64	60
Trio 2	Female	21	60	75	47
Trio 2	Female	38	57	64	50
Trio 2	Female	38	63	64	33
**Monkeys**	**Average**	**33**	**67**	**70**	**54**

**Figure 3:**
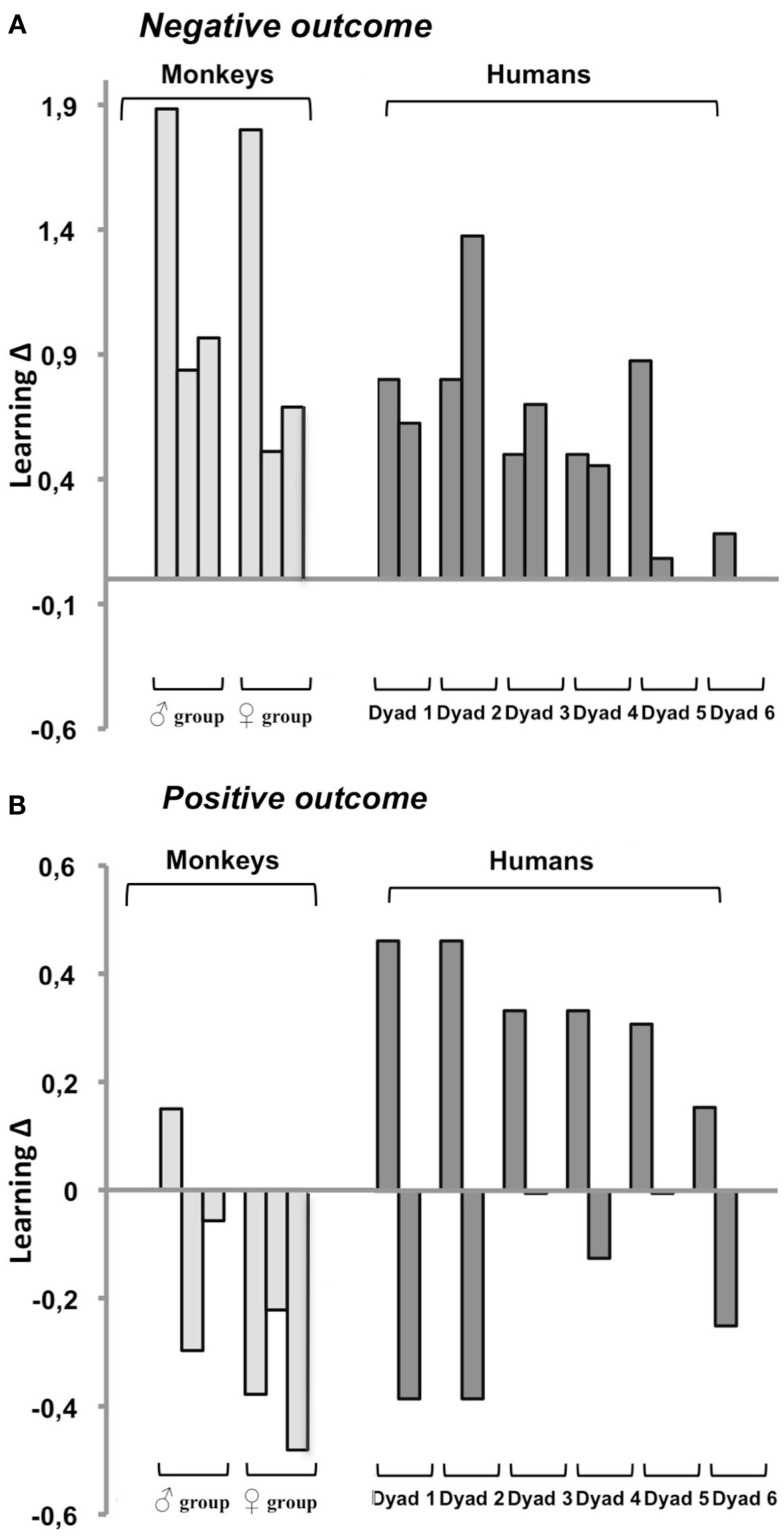
**Effectiveness of social relative to individual learning after a single negative (A) or positive (B) outcome as measured by learning Δs (social score – individual score/individual score)**. Positive Δs indicate that social learning is better than individual learning, negative Δs that it is worse. Each bar corresponds to one subject. Humans are organized per dyad as they were tested. Monkeys are grouped per trio of housemates following their rank in the hierarchy with the top ranking on the left, each monkey within a trio was tested with one or both of his/her partner according to affinities. Any given subject occupies the same position in **(A)** and in **(B)**. Note the smaller scale and greater data heterogeneity in **(B)**.

*Subjects appear in the same order as in Figures 3A,B, the top line corresponding to the leftmost bar in each graph*.

*Bold indicates the average scores for humans and monkeys*.

### Social learning

When monkeys were given the opportunity to observe another’s choice on trial 1, the direction of the outcome effect was reversed (Figure 2B; Table 1). There, the two species achieved their best performance after a negative outcome. Monkeys reached 67% correct responses and humans 78% correct responses, compared to 54 and 73% correct responses, respectively, after a positive outcome. However, this time, the outcome effect was not reliable [main effect: *F*(1,16) = 3.8, *p* = 0.07; species × outcome interaction: *F*(1,16) = 0.7, *p* = 0.41]. Neither the 13% drop observed in monkeys, nor the 5% drop observed in humans was significant (see *post hoc* comparisons in Figure 2B). Only the main effect of species reached significance [*F*(1,16) = 6.0, *p* = 0.03] as monkeys learned less from peers than humans irrespective of the outcome.

One-sample *t*-test were used to determine whether social scores were superior to the 50% chance performance one would expect on trial 2 if subjects paid no attention whatsoever to their companion’s choices and outcomes on trial 1. Monkey’s 54% social score after a positive outcome did not differ from chance (*t*5 = 0.7, *p* = 0.51), but their 67% score after a negative outcome did (*t*5 = 4.5, *p* = 0.007). In humans, both scores were well above chance (73% after a positive outcome and 78% after a negative outcome, *t*11 = 3.9, *p* = 0.002, and *t*11 = 9.0, *p* < 0.001, respectively). Subjects from both species thus did gain some knowledge from their companions albeit not trained/instructed to do so.

### Prediction 1: When free choice hinders individual learning, social learning is optimal

According to our hypothesis, negative outcome on trial 1 epitomizes situations for which social learning should be optimal; therefore, subjects from both species should show positive, or at least null, learning Δs. As illustrated in Figure 3A, this held true for all monkeys (6/6) and all humans (12/12). Overall, learning Δs averaged +0.57 in humans (range: 0–1.37) and +1.11 in monkeys (range: 0.51–1.88). Both group scores significantly differed from 0 (one-sample *t*-tests: both *t*’s > 4.7, both *p*’s < 0.006), confirming that social learning was significantly more effective than individual learning in the two species. The group scores also differed from each other (two-sample *t*-tests: *t*16 = 2.4, *p* = 0.03), as social learning superiority was more marked in monkeys than in humans.

### Prediction 2: When free choice eases individual learning, social learning is not optimal

According to our hypothesis, positive outcome on trial 1 epitomizes situations for which individual learning should be optimal; therefore, both species should show negative, or at least null, learning Δs. As illustrated in Figure 3B, this held true for 5/6 monkeys and for half of the human subjects, one per dyad. The other members of the human dyads and the remaining monkey showed unexpected positive learning Δs. In the former 11 subjects (six humans and five monkeys), learning Δs averaged −0.23, compared to +0.31 in the remaining seven subjects (six humans and one monkey). Both measures significantly differed from 0 (one-sample *t*-tests: both *t*’s > 7.4, both *p*’s < 0.001). Thus, for positive outcome, our prediction that choice-induced preference would boost individual learning and make it surpass social learning, proved correct for a majority (61%) of subjects.

### Personal origin of inter-individual variability after a negative outcome

For negative outcome, the 18 subjects were distributed along a continuum between two extremes: one human for whom social and individual learning were equally effective (0Δ in Figure 3A, dyad 6) and one monkey for whom social learning was three times better than individual learning (+1.88Δ in Figure 3A, male group). A subject’s position along this continuum was determined by his/her personal learning skills. Indeed, learning Δs were tightly correlated with individual and not with social learning scores. This held true for monkeys (*r* = −0.90, *p* = 0.01), humans (*r* = −0.83, *p* = 0.001) and the two species taken together (*r* = −0.87, *p* < 0.001). The negative correlation indicated that the greater the subject’s difficulty to correct personal errors, the more social learning surpassed individual learning.

### Social origin of inter-individual variability after a positive outcome

**Figure 4:**
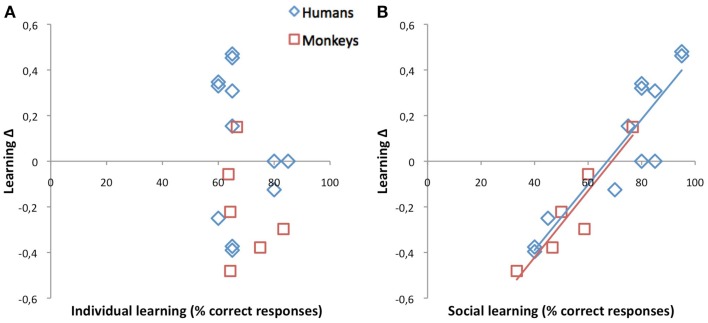
**Correlations between learning Δs, on the one hand, and individual (A) and social (B) learning scores after a single positive outcome**. How much a subject gained (positive Δ) or lost (negative Δ) by learning socially rather than individually is totally unrelated to his/her personal learning skills. For example, among the nine human subjects who reached 60–65% correct responses when learning by themselves, six gained 20–40% when learning from his/her companion, while the other three lost 20–40%. Indeed, for successes, social learning Δs were determined by social scores in both species suggesting that whether or not social learning was advantageous for a subject depended on the social dynamics within the group.

For positive outcome, inter-individual variability took the form of a dichotomy opposing 11 subjects for whom individual learning was optimal as predicted (null to negative Δs, “Δ−subject”) to seven subjects for whom, contrary to our prediction, social learning was optimal (positive Δs, “Δ+ subject”). As illustrated in Figure 4, this time, learning Δs were determined by social rather than individual learning scores (monkeys: *r* = 0.93, *p* = 0.007; humans: *r* = 0.91, *p* < 0.001; all: *r* = 0.93, *p* < 0.001). This correlation, and the fact that the dichotomy was present in each and every human dyad, suggested a social rather than a personal origin. What distinguished Δ+ subjects from their Δ− companions? Findings from the evaluations of the social dynamics within our dyads indicate that Δ+ subjects might be more dominant/aggressive than their Δ− companions.

The one Δ+ monkey was the high-ranking animal of the male group. His dominance scores stood out as he monopolized 85% of the treats during the food competition test and systematically ranked #1 on the interaction evaluation. By comparison, his counterpart in the female group secured 70% of the food and a mean rank of 1.8. In humans, the 6Δ+ subjects were not more assertive than their Δ− companions (11.7 vs. 11.2/15). They tended to be less passive (7.7 vs. 10.0/15; *t*10 = 1.8, *p* = 0.10). However, the main difference concerned their higher cumulated aggressive/manipulative scores (15.8 vs. 11.0/30; *t*10 = 2.3, *p* = 0. 04). For example, they were more likely to answer yes to the aggressive statement “I often interrupt people without realizing it on time” (5/6Δ+ vs. 2/6Δ−) and/or to the manipulative statement “I know who to see and when to see him or her, it has helped me a lot” (4/6Δ+ vs. 0/6Δ−).

## Discussion

The present study provides a controlled comparison of individual and social learning using the same object discrimination learning task in macaques and humans. Results first strengthen the idea that the phenomenon known in social psychology as choice-induced preference does affect reward-based learning in the two species. The very same monkeys and humans that, in the social situation, readily learned from their companions’ erroneous choice, had great difficulty correcting their own erroneous choice. Furthermore, the data largely confirm our hypothesis that the most effective way to learn depends on choice influence on individual learning. When choice-induced preference retarded individual learning (after a negative outcome), social learning proved optimal for all subjects. By contrast, when choice-induced preference optimized individual learning (after a positive outcome), social learning was less effective in most (though not all) subjects.

### Choice-induced preference consequences on reward-based learning

In discrimination tasks, as in many daily situations, subjects face two alternatives, one good, one bad. A single trial, whether executed or observed, should suffice to solve the problem irrespective of its outcome. A positive outcome reveals the item one should stick to, a negative one the item one should avoid. Yet, neither animals nor humans behave in this rational way. Two sets of studies have compared learning from positive vs. negative outcome in visual discrimination tasks. They reached opposite conclusions but agreed that the first outcome affects subsequent learning. Studies focused on individual learning demonstrated that humans, monkeys, cats, and pigeons learn best from (their own) successes (Riopelle et al., 1954; Riopelle, 1955, 1960; Warren, 1959; Mishkin, 1964; Itoh et al., 2001; van Duijvenvoorde et al., 2008; Cook and Fagot, 2009). Studies focused on social learning showed that macaques and birds learn best from (others’) errors (Darby and Riopelle, 1959; Templeton, 1998; see also Vanayan et al., 1985; Biederman and Vanayan, 1988). The latter phenomenon was confirmed by motor imitation studies in capuchins and human children (Want and Harris, 2001; Kuroshima et al., 2008). The present study reconciles these two sets of studies by providing a unifying hypothesis solving their apparent contradiction. We found that the very same monkey and human subjects that learn best from a positive outcome individually indeed learned best from a negative outcome socially. The first difference was marked and significant in the two species, the second milder and non-significant. The fact that choice is present in individual but not social learning could account for both differences.

Early authors (Riopelle et al., 1954; Mishkin, 1964) assumed that first-trial personal choices are dictated by preexisting attractions to one object that subsequently ease or retard individual learning depending on whether the attractive object happens to be positive or negative. Our reasoning for individual learning is similar except that we do not postulate that a spontaneous preference preexists in each subject for each and every possible pair of objects. Rather, based on the social psychology literature detailed in the Introduction, we propose that the preference that eases or retards learning is created by choice itself. The advantage of this view is that it applies to social learning as well, thereby explaining the “error paradox,” viz. why the error that is a learning handicap when personal becomes a learning aid when social.

When monkeys do not have to make the first-trial choice themselves, they simply revert to another well-known tendency, novelty seeking. This has been demonstrated long ago by Moss and Harlow, 1947; see also Mishkin, 1964; Brown et al., 1965; Deets et al., 1971; Blomquist et al., 1973). When the negative object is presented alone on trial 1, monkeys excel in avoiding it on subsequent trials because novelty seeking drives them away from already explored items. Social learning is a no-choice situation where novelty seeking may operate in a vicarious way. In sum, we propose that personal learning from errors is difficult because choice and the preference it induces for the selected item lead monkeys and humans to repeat whatever initial choice they made, even when incorrect. By contrast, social learning from errors is easy because choice absence leaves room for novelty seeking that fosters responses away from the model’s erroneous first-trial choices.

Two human neuroimaging studies (Klein et al., 2007; van Duijvenvoorde et al., 2008) indicate that learning from personal errors involves the dorsolateral prefrontal cortex, a region whose neurons carry information from one choice to the next more accurately after a positive than after a negative outcome (Histed et al., 2009). Large dorsolateral prefrontal removals leave individual learning from successes intact, selectively exacerbating monkeys’ difficulty to learn from errors (Mishkin, 1964). Interestingly, ventral lesions involving orbital areas 11/13 yield an even greater deficit raising the possibility of a link between the (physiological) perseverative tendency of healthy subjects after a personal error and the (pathological) perseveration phenomenon that has long been associated with orbital damage (Mishkin, 1964; but see Rygula et al., 2010; Bachevalier et al., 2011; Rudebeck and Murray, 2011; Noonan et al., 2012, for current qualifications). Whether selective orbital lesions would increase difficulty to learn from personal errors while sparing learning from social errors is an interesting question for future monkey lesion studies to address.

### Cross-species comparison: Macaques vs. humans

Another original contribution of the present study is the direct comparison of macaques with human adults using the same object discrimination task. The task was difficult, maximum performance reaching no more than 70–80% correct responses in either species. Difficulty was successfully equated across species by using simpler stimuli in monkeys (actual objects rather than complex patterns) and a dual-task paradigm in humans (performing the discrimination task while listening to children tales) to prevent verbal rehearsing. Subiaul et al. (2007) found an equal benefit of observation in adult macaques and 2-year old human children. Likewise, the present adult monkeys and adult humans behaved in a highly similar manner, as emphasized in the preceding and following paragraphs. Quite expectedly though, since we tested adults and not toddlers, dissimilarities did emerge.

Social learning proficiency of human adults surpassed that of macaques. This might be linked to better social attention as macaques have been reported to pay less attention to peers than chimpanzees, human children, and human adults (Rigamonti et al., 2005; Custance et al., 2006). The second cross-species dissimilarity was that monkeys’ difficulty to correct personal errors was more marked than humans’. The odds against a correct response on trial 2, already high in humans (1:1) were doubled (2:1) in monkeys. This phylogenic difference parallels the evolution described across the lifespan in both species. Learning from personal errors gradually improves from childhood to adulthood (van Duijvenvoorde et al., 2008) and then declines with aging (Itoh et al., 2001). The ability to rein in choice-induced preferences thus seems to evolve both across primate species, from monkeys to humans, and within-species, from infancy to old age. This idea fits with the finding evoked above that learning from personal errors in object discrimination tasks requires the prefrontal cortex (Mishkin, 1964), a brain region notably less developed in macaques than in humans (Passingham, 1973; Schoenemann et al., 2005), and one of the last brain regions to mature during development (Casey et al., 2000).

### Social learning superiority after negative outcome is highly reliable across species and subjects

For negative outcome, the two species displayed positive learning Δs reflecting the predicted superiority of social over individual learning. The advantage provided by social learning is remarkable in two respects: its scale and its consistency. Already impressive (+57%) in humans, the average gain was spectacular in monkeys with a twice better performance (+111%). Furthermore, not a single subject, be it monkey or human, showed a negative Δ indicative of disadvantageous social learning. Only the amplitude of the advantage provided by social learning varied (from 0 to +188%). The poorer the subject’s personal ability to correct his/her errors, the greater the superiority of social learning.

Learning from errors and related response inhibition are altered by diseases that interfere with dopaminergic function such as Parkinson’s disease (Frank et al., 2004) and Attention Deficit/Hyperactivity Disorder (Braet et al., 2011). Individual variations in these skills in healthy subjects have been linked to genetic differences in dopaminergic function in both humans (Klein et al., 2007; Frank et al., 2009) and macaques (Morgan et al., 2002; Czoty et al., 2005). In light of the present data, genetically determined poor ability to correct personal errors may have a bright side in the form of greater proficiency to learn from others’ errors.

### Individual learning superiority after positive outcome exists in both species, but not in all subjects

For positive outcome, 5/6 monkeys and 6/12 humans did present the predicted null to negative Δs indicating that they learned as well or better individually than socially. Though modest (23%), the gain provided by individual learning was significant. These data therefore proved that our dual prediction successfully accounted for the behavior of a majority of subjects (61%). This said, however, there remained an intriguing substantial minority of subjects for whom social learning unexpectedly remained superior even for positive outcome. The benefit was again modest (31%) but significant. Across all 18 subjects, learning Δs were linked to social rather than individual learning scores, suggesting that, this time, social dynamics within the actor/observer dyad were responsible for inter-individual variations. Supporting this idea was the fact that, within each and every human dyad, one subject favored individual learning, while the other favored social learning.

In monkeys, the unexpected profile of social learning superiority occurred only in the high-ranking male whose dominance was associated with a monopolization of both food and attention. In humans, subjects self-assessed their social styles using a scale measuring assertive, passive, aggressive, and manipulative behaviors. Manipulation being an indirect form of aggression (Hess and Hagen, 2006), scores for the latter two categories were cumulated. Though crude, this self-assessment pinpointed a potential characteristic associated with high social learning proficiency. Namely, the unexpected social learning superiority generally occurred in the member of the pair with the stronger tendency to resort to direct and/or indirect aggression in daily-life. This finding is reminiscent of that seen in pairs of chimpanzees, mangabeys, and rhesus macaques, in which the dominant individual exploits the activities of the subordinate (Menzel, 1973; Coussi-Korbel, 1994; Drea and Wallen, 1999). High rank confers substantial social advantages in many primates including humans (Shively, 1998; Sapolsky, 2005; Zink et al., 2008; Ly et al., 2011), the present data suggest a link between dominance and social learning proficiency that certainly deserves to be further explored.

### Could factors other than choice absence explain why it is so much easier to learn from others’ errors than from personal ones?

At least three factors differ between social and individual learning from errors: choice (missing vs. mandatory), but also action (observed vs. executed), and failure to obtain a reward (vicarious vs. experienced). That observed action and vicarious feedback could be more efficient than executed action and experienced feedback is counterintuitive (Bandura, 1977). In addition, there is no evidence that the brain codes others’ action or errors more effectively than one’s own. The primate brain does differentiate others’ action from self-action (Yoshida et al., 2011) and others’ failure to get a reward from one’s own (Yu and Zhou, 2006; Bellebaum et al., 2010; Burke et al., 2010). Yet, there is massive evidence that observed and executed actions share the same neural code (mirror neurons, e.g., Bonini and Ferrari, 2011) as do vicarious and experienced negative feedback (error/feedback-related negativity, van Schie et al., 2004; Yu and Zhou, 2006; Shane et al., 2008). We confirmed this in a recent fMRI study showing that the same parieto-frontal brain network subserves stimulus-response associations whether they were acquired via social or individual feedback-based learning (Monfardini et al., 2008).

Choice therefore seems to be the main culprit behind the huge difference in social and individual learning effectiveness after a single negative outcome. O’Doherty (2004) and Bellebaum et al. (2012) demonstrated that striatum activation during reinforcement learning tasks depend on whether or not subjects have to perform a response to receive reward. The literature on choice-induced preference goes further by affirming that commitment to a stimulus alters its hedonic value, a change accompanied by a post-decision increase of caudate activation for the selected option, and a decrease of caudate activation for the rejected ones (Sharot et al., 2009; Izuma et al., 2010). This means in our protocol a revaluation of the object that will, only moments later, prove unrewarded. The present behavioral data suggest that this early choice-driven value change can be powerful enough to totally obliterate the subsequent outcome-driven value change. Research in neuroscience has heretofore focused on bottom-up modulation of value by decision outcome (reinforcement learning), choice-induced preference is an example of top-down cognitive modulation of value based on decision itself.

### Social learning as a way to overcome poor capacity to learn from one’s own errors?

Examples of how humans and other animal species fall prey of their own errors are not limited to reinforcement learning, several have been reported by psychology and neuroeconomics. The Concorde fallacy (also known as the sunk-cost effect) is one we share with lower animals such as wasps and pigeons. Named after the supersonic airplane to refer to hopeless endeavors that we keep pursuing because we have already invested too much in them to quit, this irrational persistence operates only in those involved in the initial decision (Navarro and Fantino, 2005). More specific to humans are the action-observer bias, the tendency to attribute others’ failures to their personality, and one’s own failures to the situation (Jones and Nisbett, 1971), and the optimistic bias, the systematic tendency to overestimate the outcome of our actions (Sharot et al., 2011). All of these biases indicate that the value of an option is not always rationally updated after a negative outcome. Thus, seeing the world from one’s sole perspective does not always allow to reliably tell good from bad. By providing us with a different perspective, social leaning could be a way to counterbalance the shortcomings of personal valuation processes.

## Conclusion

Social learning is currently receiving much theoretical attention. Yet, too few controlled studies have compared the usefulness of the exact same amount information acquired individually vs. socially to obtain an accurate empirical picture of the factors that determine the best way to learn. The present study identifies one of these factors, namely, choice and the preference it is known to induce for selected options. It thus reinforces models emphasizing social learning benefits. Indeed, in addition of saving effort and time and allowing knowledge accumulation over generations, social learning could also be a protection against potentially harmful personal biases. Social learning nevertheless has its limits and these limits may vary across subjects depending on their social characteristics.

## Conflict of Interest Statement

The authors declare that the research was conducted in the absence of any commercial or financial relationships that could be construed as a potential conflict of interest.
